# Lateral Flow Assay to detect CXCL10 as a diagnose-supporting biomarker for Polyomavirus-associated Nephropathy (PVAN) after kidney transplantation (KTx)

**DOI:** 10.1038/s41598-026-66553-7

**Published:** 2026-08-27

**Authors:** Emely Hauch, Lisa K. Seiler, Marianne Hoyer, Irina Scheffner, Wilfried Gwinner, Patrick Lindner, Cornelia Blume

**Affiliations:** 1 Institute of Technical Chemistry, Leibniz University Hannover, Callinstraße 3-5, 30167 Hannover, Germany; 2 Department of Hypertensiology and Nephrology, Hannover Medical School, Carl- Neubert Straße 1, 30625 Hannover, Germany

**Keywords:** Biomarkers, Diseases, Medical research, Nephrology

## Abstract

Kidney grafts, as the most transplanted organs, require intensive monitoring to ensure optimal transplant survival rates for patients. These rates can be jeopardized by severe, difficult-to-treat opportunistic infections such as polyomavirus, which can lead to polyomavirus-associated nephropathy (PVAN). There is an urgent need for non-invasive biomarker tests to narrow down the number of patients who require a transplant biopsy to confirm PVAN. The chemokine CXCL10 was significantly elevated in urine samples from kidney transplant recipients (KTR) with biopsy-confirmed PVAN compared to KTRs with a protocol biopsy found to be normal (114.8 ± 177.3 pg/ml, *n* = 22 vs. 11.4 ± 22.3 pg/ml, *p* < 0.0001, *n* = 45). Therefore, we developed a lateral flow assay (LFA) for the detection of PVAN with an area under the curve (AUC) of 0.832 according to receiver operating characteristic analysis as a point-of-care test.

**Supplementary Information:**

The online version contains supplementary material available at https://doi.org/10.1038/s41598-026-66553-7.

## Introduction

Contrary to the growing number of patients with end-stage renal disease, the number of available transplants is scarce. Additionally, the success of kidney transplantation (KTx) is threatened by infections and rejections^1^. One of these complications is represented by a polyomavirus-associated nephropathy (PVAN), induced by the BK polyomavirus (BKPyV). This virus is found in up to 90% f the population without any clinical significance^2^, but becomes active under immunosuppression. PVAN in kidney transplant recipients (KTRs) thus has an incidence of 1–10% after KTx. The latent BKPyV can be reactivated in the KTR or freshly transferred to the KTR from the donor.

Since the immunosuppression is usually most intense in the first year after KTx, the PVAN rate is highest during this period^3^. The first measure to lower the BKPyV load is to reduce the dosage of immunosuppressives to the minimum acceptable level, carefully balanced against the ongoing threat of rejection. No curative therapy is present, since the effect of the antiviral cidofovir with nephrotoxic side effects is not proven, the benefit of leflunomide and immunoglobulins is questionable^4,5^, and virus-specific T-cell therapy is still in its infancy^6^.

PVAN, going along with tubulointerstitial nephritis and subsequent graft fibrosis threatens with graft failure and following rejection due to a lowered immunosuppression, and thus many centres perform a renal graft biopsy with specific immunohistochemical findings such as SV 40 antigen staining for confirmation. According to BANFF, there are three stages of PVAN^7^ delineating acute inflammation and the grade of fibrosis. Since a biopsy is an invasive measure, reliable noninvasive tests to strengthen the diagnosis PVAN, relying on urine biomarkers are urgently needed^8,9^. Early urine biomarkers before the full-blown picture of e.g. a PVAN is overt in a biopsy can support clinical decision making of how to guide immunosuppressive therapy^10,11^. There are already such tests, for example the kSORT gene panel, supporting the diagnosis of a chronic rejection^12^. The complex and costly testing of cell-free DNA in blood can predict rejection with some limitations due to positivity in cases with non-specific tissue injury^13^.

A recent prospective study with protocol biopsies presented the chemokine CXCL10 as blood and urine indicator of PVAN in 85 KTRs with various stages of BKPyV replication^14^. Here, CXCL10 levels were low before BKPyV detection, increased with low-level DNAemia, and further rose as DNA loads exceeded 1000 copies/ml, despite no PVAN evidence. Peak levels occurred when BKPyV-DNAuria and DNAemia were obvious, accompanied by clinical or histological PVAN signs. Another recent study focused on urine CXCL10 levels increasing to 16.42 ng/mmol in KTRs in parallel to viruria and decoy cells, with a maximum of 42.5 ng/ml in KTRs with additional DNAemia^15^. Thus, CXCL10 can be suggested as an early biomarker for urine monitoring in PVAN.

CXCL10 is a γ‑interferon–induced chemokine that binds CXCR3 on immune cells and attracts Th1 lymphocytes, monocytes, and NK cells to amplify inflammation. This marker can also be observed in autoimmune disease, glomerulonephritis, or atherosclerosis, and it is considered a strong biomarker after kidney transplantation due to its detectability even under immunosuppression^16–21^.

In this work, we built up a Lateral Flow Assay (LFA) as point-of-care-test (POCT) for kidney transplanted patients with suspected PVAN and also tested it in subgroups of KTRs with different rejection types. An LFA detects target substances in liquid samples without specialized equipment, using capillary beds for fluid transport and reaction zones for molecule interaction. Test and control lines confirm detection, while excess fluid is absorbed. An LFA thus is a fast, reliable method for KTR monitoring, possibly prompting biopsy verification and intervention by the nephrologist to adjust immunosuppression or monitor the resolution of the PVAN after biopsy if necessary^5^.

In the work presented here, we used a panel of urine samples out of a representative biobank, established with protocol and indication biopsy patients at Hannover Medical School^22^. The test performance of the LFA for CXCL10 was investigated using receiver operating curves (ROC).

## Results

### CXCL10 concentrations in different diagnostic entities according to the graft biopsy

CXCL10 levels (0 to 832.8 pg/mL) and creatinine values (0.0 mg/dL and 249.5 mg/dL) were determined in a total of 119 urine samples using ELISA.

**Fig. 1:**
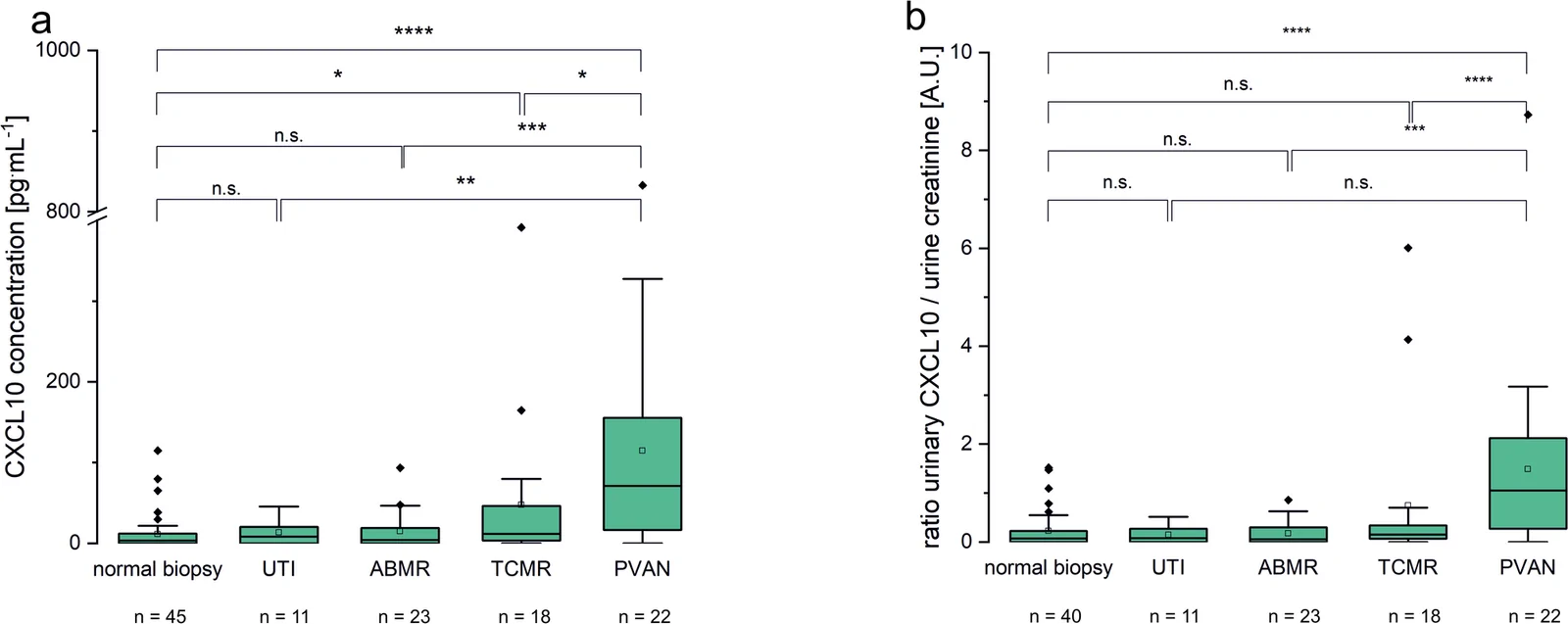
Boxplots of CXCL10 urine concentrations in samples of KTRs with a normal biopsy, urogenital infection (UTI), antibody-mediated rejection (ABMR), T cell-mediated rejection (TCMR), or polyomavirus-associated nephropathy (PVAN). **(a)** rsp. absolute CXCL10 levels of these different entity groups plotted against the CXCL10/creatinine ratios **(b)**. Diagram (a) shows absolute concentration, and (b) shows the ratio of CXCL10/creatinine in urine samples of kidney transplant recipients (no concentration of creatinine available for five patients). Asterisks indicate the level of significant differences between certain compared sample groups (*p < 0.05, **p < 0.01, ***p < 0.001, ****p < 0.0001 according to the Mann-Whitney-U-test, n.s. = not significant), with samples grouped according to their biopsy finding as indicated. Quartiles were calculated using the Tukey method. The boxes are showing the interquartile range (IQR) from Q1 (25th percentile) to Q3 (75th percentile), with the median (50th percentile) shown as a line within the box. The whiskers extend up to 1.5 times the IQR; outliers beyond this range are shown as individual data points. Means indicated in square points.

CXCL10 concentrations in urine from KTRs with PVAN (*n* = 22; 17 protocol, 5 indication biopsies, median = 71.2 pg/mL, range 16.6–149.9, see Fig. 1a) were significantly higher than in all other KTR groups examined. The level of significance was *p* < 0.0001 vs. KTRs with a normal biopsy (*n* = 45, 43 protocol, 2 indication biopsies, 3.5 pg/mL; 0.00-11.9), whereas it was *p* < 0.001 vs. KTRs with antibody-mediated rejection (ABMR) (*n* = 23, 15 protocol, 8 indication biopsies, 4.0 pg/mL; 0.2–19.0) and still significant with *p* < 0.01 vs. KTRs with a normal biopsy but urinary tract infection (UTI) (*n* = 11, 10 protocol, 1 indication biopsies, 8.2 pg/mL,0.0-20.1) and with *p* < 0.05 vs. KTRs with a T-cell-mediated rejection (TCMR) (*n* = 18, 14 protocol, 4 indication biopsies, 11.5 pg/mL, 3.5–43.4). CXCL10 levels in the urine of KTRs with TCMR were also significantly higher than in KTRs with a normal biopsy (Fig. 1a). We also assessed the CXCL10/creatinine ratio in all these KTR groups, and the comparative analyses showed the same tendency (Fig. 1b).

CXCL10 levels correlated with the CXCL10/creatinine ratio (Fig. S 2), indicating no impact of urine dilution grade or secretion/reabsorption processes in the tubular system on chemokine excretion (R^2^ = 0.89). When analyzing only patients with protocol biopsy (the majority in each KTR group), we found no significant changes in the results (Fig. S 3, Fig. S 4). Further information on KTRs is given in Table 2 (method section).

### CXCL10-LFA tested with patient urine samples

**Fig. 2:**
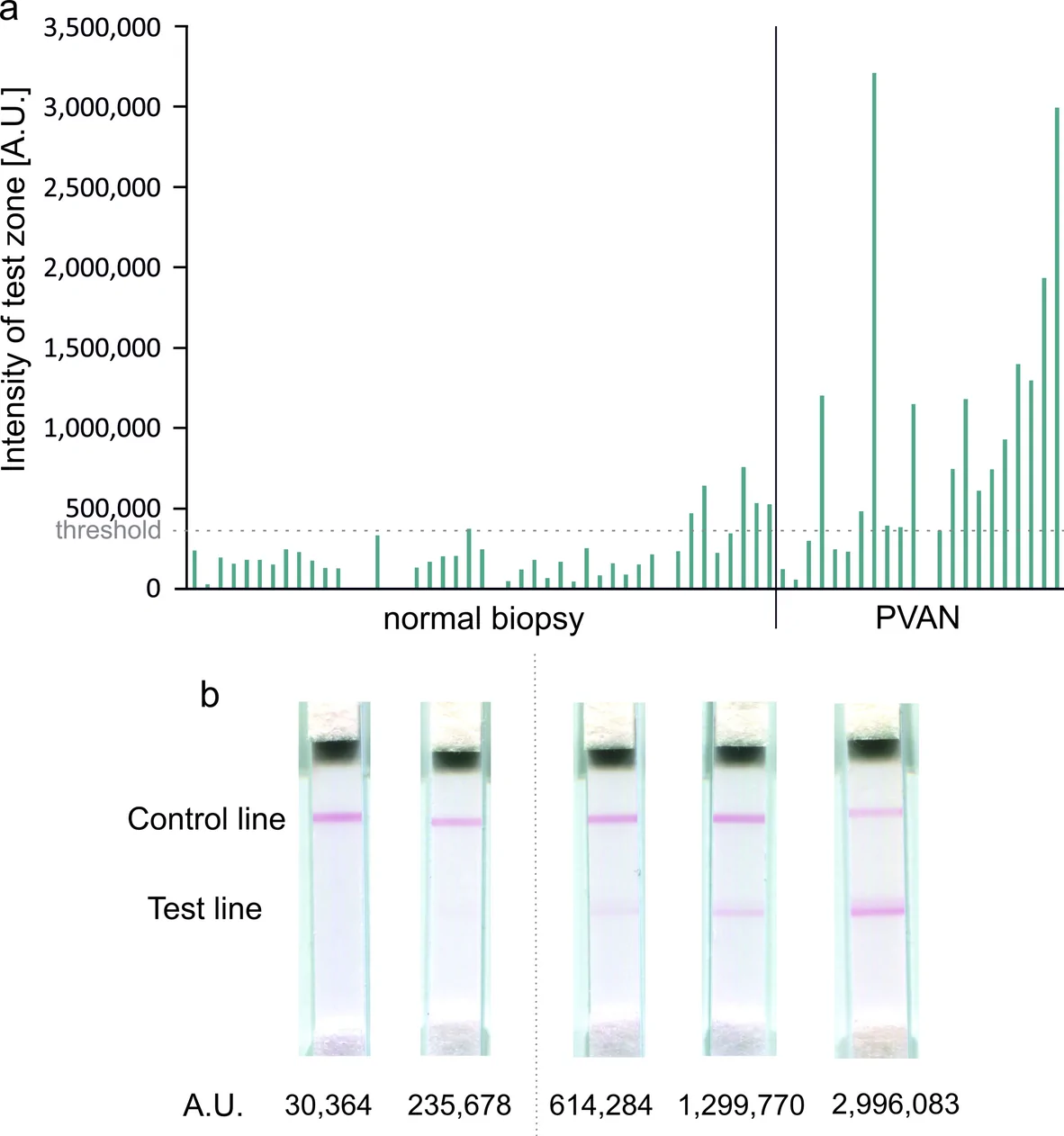
LFA band intensities, depicted as column diagrams reflecting the test signal of samples with a normal biopsy and with Polyomavirus-associated nephropathy (PVAN). The respective values **(a)** were determined using scans (shown as an example in **b)** and ImageJ results of the Lateral Flow Assay strips with patient samples showing the test zone (TZ) and control zone (CZ). Afterwards, Receiver operating characteristic curves were performed to define the threshold (363870 AU). **(b)**The bands represent two negative Lateral Flow strips below the defined threshold and three positive Lateral Flow strips of varying intensities above the threshold.

The column diagrams corresponding to the distinct LFA stripes show the scan intensity of the test zone measured with ImageJ and document the test results of the patient samples. All samples applied on the LFA showed a positive result for the control zone, indicating that the run-through and test performance were appropriate. To avoid unambiguous test results, a threshold of 363,870 A.U. was defined by performing ROC, with all 22 patient samples with biopsy-proven PVAN set as positive results, and all 45 patient samples classified as normal by biopsy set as negative. All LFAs with an applied sample were scanned, and the red color lines were analyzed with ImageJ (Fig. 2). LFAs with a signal intensity of the test zone lower than 363,870 A.U. were defined as negative. BK viral load in serum (given in copies per mL) and urinary CXCL10 concentrations in confirmed PVAN-positive patients showed no significant association (Spearman´s *r* = 0.13, *p* = 0.59). No association was found between rising CXCL10 concentrations and a higher PVAN class (Spearman´s *r* = 0.04, *p* = 0.86), and there was no significant difference in CXCL10 concentrations across the three different biopsy classes (Fig. S 5).

**Fig. 3:**
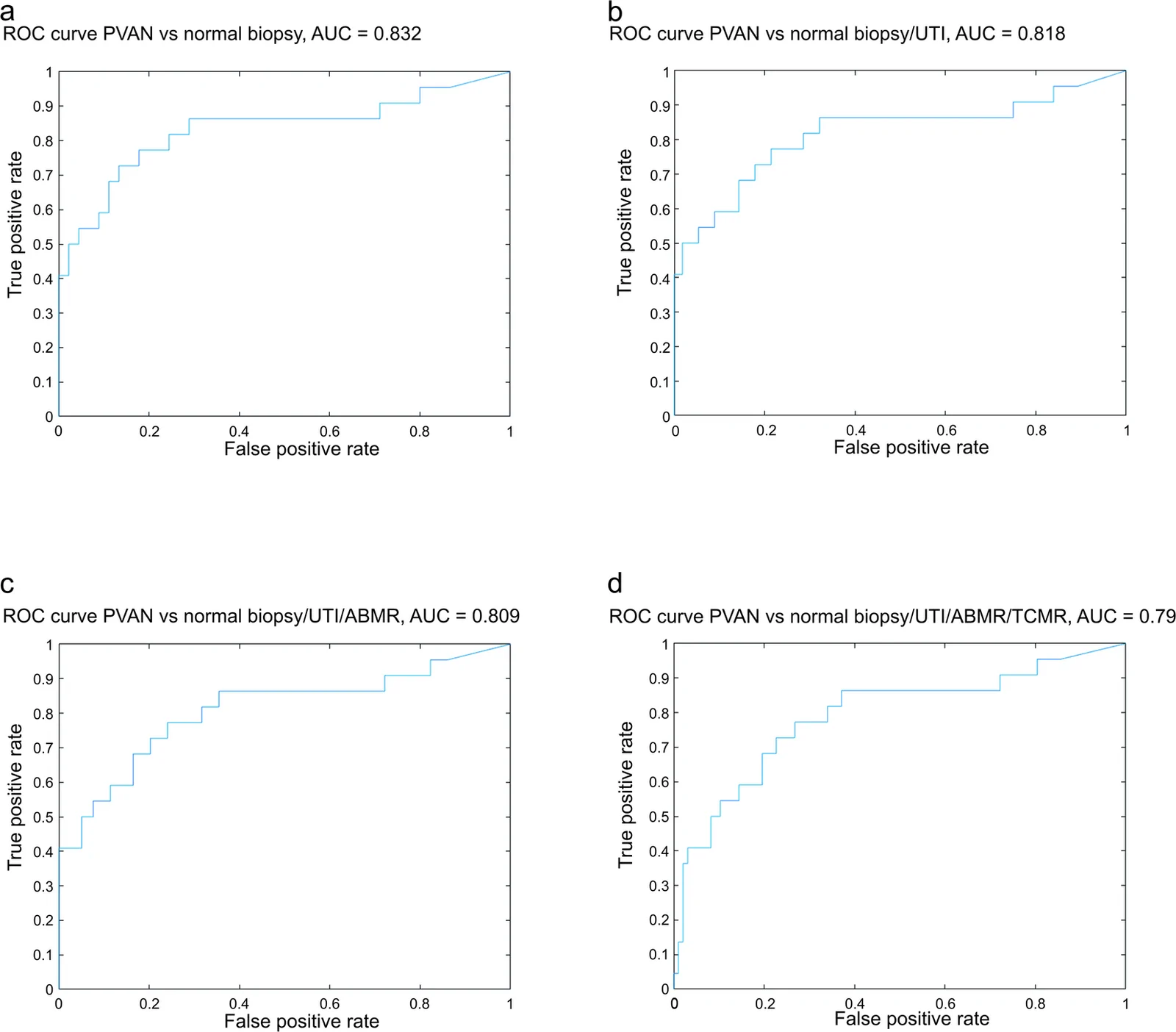
Receiver Operating Characteristic (ROC) Curves, describing the test performance of the newly developed lateral flow assay. The ROC curves comprise LFAs with samples from patients diagnosed with Polyomavirus-associated nephropathy (PVAN, *n* = 22) according to BANFF criteria. **(a)** Samples from patients with no histological findings according to BANFF criteria (normal biopsy, *n* = 45), **(b)** additionally with samples from patients with urinary tract infection (UTI, *n* = 11), and **(c)** additionally with samples from patients with antibody-mediated rejection (ABMR, *n* = 23). **(d)** All 18 samples from patients with a T cell-mediated rejection (TCMR) were used.

Figure 3 shows the respective areas under the curve (AUC) results from the ROC analyses. LFAs of PVAN patients vs. LFAs from KTRs with a normal biopsy showed an AUC of 0.832 (Fig. 3a). The AUC of testing samples from PVAN-KTRs vs. samples from KTRs with a normal biopsy but UTI was in the same range (AUC of 0.818, Fig. 3b). The test was also discriminatory for samples of PVAN-KTRs vs. samples of KTRs with a normal biopsy, UTI, or an ABMR (AUC of 0.809, Fig. 3c). The ROC curve of PVAN-KTRs vs. KTRs with a normal biopsy, UTI, ABMR, or TCMR showed an AUC of 0.79 (Fig. 3d). The ROC analyses also showed AUC values of 0.789 in PVAN KTRs vs. those with ABMR (Fig. S 6a) or 0.708 in PVAN KTRs vs. those with TCMR (Fig. S 6b).

The characteristic parameters of the LFA are listed in Table 1 as determined by ROC.

**Table 1:** Listing of the different sensitivity, specificity, negative predictive value (NPV), positive predictive value (PPV), and area under the curve (AUC) when comparing lateral flow assays with various histological findings (PVAN = polyomavirus-associated nephropathy, UTI = urinary tract infection, ABMR = antibody-mediated rejection, TCMR = T cell-mediated rejection). Threshold levels were determined by ROC.

	PVAN vs. normal biopsy	PVAN vs. normal biopsy/UTI	PVAN vs. normal biopsy/UTI/ABMR	PVAN vs. normal biopsy/UTI/ABMR/TCMR
Sensitivity [%]	71.4	91.7	100	80.0
Specificity [%]	84.8	83.3	85.9	87.2
NPV [%]	86.7	98.2	100	97.9
PPV [%]	68.2	50.0	40.9	36.4
AUC [A.U.]	0.832	0.818	0.809	0.790
Threshold [A.U.]	363,870	745,941	931,770	1,153,376

## Discussion

Finding a new method for the follow-up examinations for kidney transplant patients that is fast, simple, non-invasive, and does not require creatinine measurement would be highly beneficial. As outlined before, the BK polyomavirus persists for life in a non-replicative, latent form, primarily in tubular and urothelial cells and potentially reactivates under immunosuppression^23^; in such cases, viruria proceeds viremia^4^. Urine-based tests (PCR indicating BKV viral load or cytological detection of so-called decoy cells^24^ vote for replicative activity and support the assessment of suspicion and monitoring; however, they do not replace either standardized virological diagnostics in plasma or histological confirmation of PVAN via biopsy. According to current clinical guidelines, immunosuppression may be adjusted based solely on sustained BK viremia, even without allograft biopsy. In this specific situation, an additional diagnostic measure, such as the CXCL10 LFA presented here is a diagnostic-supporting measure since the test indicates ongoing tubuloepithelial inflammation. The Banff classification for PVAN provides a standardized semiquantitative staging system, taking into account both the tissue viral load (*pvl* score), but also the extent of chronic injury (*ci* score), which imparts substantial prognostic value. In addition, a biopsy can also reveal concurrent pathologies such as rejections particularly in the context of reduced immunosuppression in response to rising BKV viral load in the serum^7,25^. Neither BK viral replication nor CXCL10 do always equate to histologically detectable nephropathy. This may be plausible in later PVAN stages, since fibrosis, which is a correlate of tissue damage and scarring, predominates. We consider the value of the CXCL10 – LFA as an early test to decide upon the severity of the BKV-induced inflammation and on the necessity of a biopsy. As such, the LFA for detecting CXCL10 may be a suitable tool to complement existing methods and serve as a POCT in the hands of KTRs^26^.

This marker was previously also proposed as an indicator of rejection^19,27,28^. As evaluated by a standard ELISA, CXCL10 in our work was only moderately elevated in samples of patients suffering from TCMR in contrast to those with PVAN. We suggest that this was because samples used here derived predominantly from KTRs with a protocol biopsy revealing unexpected cases of TCMR with comparably lower severity and inflammation, not reflected by a rise of serum creatinine, and representing rather early states of rejection^29^. This is in line with the observation that high CXCL10 concentrations in outlier samples of patients with TCMR correlated to a higher degree of severity of the tubulointerstitial rejection. Importantly, the CXCL10 result was not confounded by urinary tract infection present in 20% of the control samples without rejection or PVAN, similar to findings by Horvanth et al.^30^. The robustness of the CXCL10 analysis in conditions with UTI is advantageous, as it is frequently present in kidney transplant patients.

We suggest that CXCL10 occurs at different levels in different pathological states of the renal graft. This can be because CXCL10 secretion varies by cell type. In PVAN, tubular epithelial cells are affected by the virus infection and produce the marker. In rejection, endothelial cells probably generate the chemokine after cross-talk with immunocytes such as activated T cells (e.g. in TCMR) or B-cells (producing donor-specific antibodies, e.g. in ABMR).

Elevated levels also correlate with renal disease severity, reflecting production in mesangial cells, podocytes, or epithelial cells post-transplant. It is assured that CXCL10 indicates (sub-) clinical rejection and is linked to tubulointerstitial inflammation in PVAN^31–34^. However, the BANFF stages of PVAN in the KTRs analyzed in this work did not clearly correlate with CXCL10 levels. We suppose that here KTR numbers, particularly in PVAN class 3, were too low. For this reason, the patient group should be expanded in a future prospective study, primarily to examine the differences among the respective PVAN classes in greater detail and to correlate them with CXCL10 levels. The trend observed so far – that CXCL10 concentration does not correlate with PVAN class – can nevertheless be explained, since CXCL10 acts as an early marker for tubuloepithelial inflammation and fibrosis predominates, particularly in the later stages of PVAN. For this reason, it is entirely possible that even higher PVAN class does not necessarily lead to an increase in CXCL10 concentration.

ROC analysis of the LFA presented in this work showed an AUC as high as 0.832 in PVAN-KTRs vs. those with a normal biopsy or 0.818 in PVAN-KTRs vs. those with a normal biopsy and an UTI or 0.809 in PVAN-KTRs vs. those with a normal biopsy or UTI or an ABMR. Even in a direct comparison between PVAN KTRs vs. those with ABMR we showed an AUC of 0.786 or 0.708 in PVAN KTRs vs. those with TCMR. This means that the marker is a good distinguishing criterion between these different named kidney transplant conditions. We see a certain limitation in this work, since we also saw a significant difference between CXCL10 levels of PVAN-KTRs and those with a TCMR, but we consider the TCMR patients analyzed here as a specific group with low to moderate rejection activity in their biopsies. Thus, this needs re-testing in a prospective study to evaluate whether the limit of detection of CXCL10 in the urine of KTRs with either PVAN or TCMR is distinctively different or not.

These LFA performance results show that this test platform is suitable for the simplified detection of PVAN. For the evaluation of the LFA in this work, we used image processing software (ImageJ). As a further approach to raising the accuracy of the LFA test result, prospectively, a test device could be used for automated test evaluation. LFA images could be processed by suitable application software (app). The app could immediately indicate the test result (positive/negative) as shown by others before^35,36^. Using an app with a defined cut-off for CXCL10 could prevent misinterpretation, especially in the grey zone between a normal CXCL10 concentration and a pathological CXCL10 concentration indicating PVAN^37–39^.

We consider the LFA developed in this work as a valuable diagnostic tool for Tx outpatient clinics, wards, or large outpatient nephrological departments. While not a biopsy substitute, LFA can refine diagnoses and reduce unnecessary biopsies for complications like PVAN.

## Conclusion

The newly developed LFA detecting CXCL10 for diagnosing PVAN after Tx reaches a sensitivity (71.4%) and specificity (84.8%) with an AUC of 0.832 (PVAN vs. normal biopsy), but the study is limited by the use of cryopreserved urine samples. Since this is a single-center, retrospective study, we thus suggest to conduct a longitudinal prospective study. This will make it possible to determine whether the test can detect the disease even before obvious PVAN symptoms appear in freshly taken urine without the danger of chemokine degradation. In addition, further studies must assess in which concrete period after the suspected diagnosis of a PVAN, maybe according to lowered graft function and an increased viral load, the CXCL10-LFA is useful. After a positive test, patients should consult a transplant physician for biopsy confirmation and medication adjustment. Nevertheless, our work confirms CXCL10 elevation in PVAN-affected KTR urine, supporting its use as a POCT biomarker for an LFA. Polyoma-specific PCR remains essential for diagnostic certainty. A multi-center study is needed to address single-center limitations and for clinical transfer of the LFA, possibly also in post-biopsy monitoring.

## Materials and methods

### Patient sample collection and ethics vote

Urine samples taken at the time of a protocol biopsy or an indication biopsy (99 vs. 20) from the biobank of the Department of Nephrology in Hannover Medical School were utilized. The patients - aged between 18 and 74 years - were transplanted between the years 2008 and 2015 and had regular follow-up outpatient visits at the transplant center. Samples and patient data were taken according to the guidelines of the Helsinki Declaration and approved by the ethics committee at Hannover Medical School (Ethical approval Hannover Medical School no. 7370) after informed consent. Urine sampling occurred immediately before the graft biopsy, and samples were cryopreserved at -80 °C as described earlier^40^. The sample collection comprised samples from 18 patients suffering from TCMR, 23 patients with ABMR, 22 patients with a histological finding of a biopsy-proven PVAN, and 45 patients with a normal biopsy (= no rejection, no urinary tract infection, no PVAN). Biopsies were histomorphologically scored using the BANFF classification version valid at the time of biopsy^41^. Furthermore, 11 patients had a diagnosis of a UTI according to urine analysis (i.e., leukocyturia and a positive bacterial urine culture) at the time of urine sampling. Further details of the analyzed patients are given in Table 2 and Table S1. Among the 22 patients with a histological finding of a biopsy-proven PVAN, BKV replication was detected in the serum of only 21 patients. In 20 of these patients, BKV replication was detected to a varying degree (median = 34000, range 85000–505451). The one patient tested negative for BKV in the serum, was tested clearly positive for the JC virus (34000 copies per mL at the time of the index biopsy). Further details on the histological PVAN classes, as well as the respective BKV loads and CXCL10 concentrations are set out in Table S2. The classification of the PVAN-KTRs was performed according to BANFF criteria^7,42^.

**Table 2:** Characteristics of the kidney transplant recipients with numbers. Sex, age, transplant age, delayed graft function, immunosuppressive regimen, history of rejection or re-grafts, protocol or for cause biopsy, and period after Tx in months. Abbreviations: CyA = cyclosporine A, Tac = tacrolimus, MMF/MPA = mycophenolate mofetil or mycophenolic acid, SIR = sirolimus.

	Non-rejection	TCMR	AMR	PVAN	UTI
**Females**	20/45	5/18	15/23	6/22	10/11
**Age**	47.9 ± 14.8	50.0 ± 13.5	48.4 ± 8.8	51.7 ± 14.9	54.1 ± 16.7
**Donor age** **(year)**	53.1 ± 10.9	57.9 ± 13.0	44.9 ± 13.8	58.1 ± 12.9	61.7 ± 17.9
**Donortype** **Deceased** **Living: related** **Living: unrelated** **Unknown**	23/45 10/45 4/45 8/45	15/18 1/18 2/18	16/23 5/23 2/23	11/22 6/22 5/22	9/11 2/11 0/11
**Delayed graft function**	7/45	5/18	3/23	2/22	2/11
**Immuno-** **suppressive drug regimen**	15 Cya 30 Tac 43 MMF/MPA 0 SIR 45 steroids	8 Cya 9 Tac 16 MMF/MPA 1 SIR 17 steroids	4 Cya 19 Tac 22 MMF/MPA 3 SIR 23 steroids	1 Cya 19 Tac 16 MMF/MPA 5 SIR 22 steroids	2 Cya 9 Tac 9 MMF/MPA 2 SIR 11 steroids
**Previous rejection**	14/45	10/18	18/23	10/22	3/11
**Re-transplants**	5/45	2/18	6/23	1/22	0/11
**Protocol biopsy**	43/45	14/18	15/23	17/22	10/11
**Biopsy after KTx (months)**	7.2 ± 12.9	7.8 ± 7.5	15.3 ± 19.5	7.1 ± 6.4	4.2 ± 3.2
**Serum creatinine levels at day of Biopsy (median)**	136 ± 38.8	161.5 ± 85.1	123 ± 87.9	180.5 ± 56.9	144 ± 53.3

### Determination of CXCL10 and creatinine concentration in urine samples

Urinary CXCL10 concentrations were first determined using the human CXCL10/IP-10 DuoSet ELISA (R&D Systems, Cat# DY266-05), and creatinine concentrations in urine were measured by the creatinine parameter assay kit (R&D Systems, Cat# KGE005), according to the manufacturer’s instructions. The ratio of CXCL10/creatinine was determined as a dimensionless key figure to test the possible impact of urine dilution grade or secretion/reabsorption processes in the tubular system on chemokine excretion.

### Materials for the LFA

An LFA is based on a capture and detection antibody raised against the analyte CXCL10 and on an unspecific anti-IgG-antibody to test the run-through performance of the LFA (Fig. S 1). Thus, on the test line, the antibody MAB 2661 from R&D Systems (Minneapolis, USA) and on the control line, a goat anti-mouse from Meridian Life Science (W41502G, Tennessee, USA) were applied as a capture antibody against CXCL10. The protein ABIN2748997 (ABIN2 in the following) was used as a detection antibody against CXCL10, and CXCL10 was used as a spiking sample (both from Antikörper online, Aachen, Germany).

The absorbent pad (grade 222) and sample pad (grade 6615) were obtained from Ahlstrom-Munksjö (Helsinki, Finland). The nitrocellulose membrane was kindly provided by Sartorius Stedim Biotech (Sartorius Unisart^®^ CN180 backed, Goettingen, Germany). Fassisi GmbH (Goettingen, Germany) kindly provided the gold nanoparticles (AuNPs). Tween20 was purchased from PanReac AppliChem (Darmstadt, Germany) and NaCl from Sigma-Aldrich Chemie GmbH (München, Germany). Phosphate-buffered saline (PBS), sodium borate buffer, Tris, and the running buffer were prepared as described in Seiler et al.^43^.

### Preparation and Assembly of the Lateral Flow Assay

For the LFA, a sample pad, membrane, and absorbance pad were stuck to the backing card and cut into strips of 4 mm (Cutting machine Dahle 562 m Dahle, Ems, Germany). Before, the sample pad was pretreated with Tris (100 mM, pH 8.0) for 4 h. The capture antibodies on the test and control zone were applied by hand using a volume of twice five drops à 0.1 µL in a line, and dried at RT for at least four hours. To achieve stable conjugates of detection antibody and gold nanoparticles, a flocculation test was used that allows for determining the appropriate ratio of antibodies per AuNP as described earlier^40^; here, a detection antibody concentration of 6.96 µg/mL was identified.

In preliminary tests, the optimal conditions (membrane coated with 2 µL/cm (0.5 mg/mL) capture antibody, conjugate: 1 µL, pre-incubation: 15 min) for the most intense test signal were evaluated: For each LFA run, an amount of 100 µL patient urine sample was incubated for 15 min at RT with 1 µL of AuNP-ABIN2. The strip was placed in the pre-incubated sample. After another 15 min, the strip was scanned (Epson Perfection V370) and the scan was analyzed using ImageJ to receive intensity histograms of the positive signals (red lines).

### Statistics

After performance of the Shapiro-Wilk test to exclude normal distribution, significant differences concerning the CXCL10 levels in the urine samples were evaluated using the Mann Whitey U test (with *p < 0.05, **p < 0.01, ***p<0.0.001, ****p<0.0.0001). Spearman´s rank correlation was used to calculate the correlations between the BKV replication/PVAN classes and CXCL10 concentrations. A Kruskal-Wallis ANOVA was performed to assess the differences between the individual PVAN classes and their CXCL10 concentrations. Receiver operating characteristic curve analyses were performed to display the AUC using MATLAB R023a with `perfcurve`. By using this command, it was possible to test all threshold values and identify the optimal point at which the sum of the errors is smallest. For the calculation, all 22 patient samples confirmed as PVAN by biopsy were classified as positive, and all 45 patient samples confirmed as normal by biopsy were classified as negative. The lateral flow assays run with patient samples were scanned, and the intensity of the test and control lines was determined using ImageJ-win64. Through this analysis, an intensity value for the test line (A.U.) was assigned to each patient sample. ROC analyses revealed a false positive rate of 0.171, a true-positive rate of 0.696 and the LFA threshold of 363,870 A.U. Sensitivity and specificity were defined according to a binary classification test for CXCL10 using the signals of the LFA. Negative predictive value (NPV) and positive predictive value (PPV) were calculated using the formula

$$\:\mathrm{N}\mathrm{P}\mathrm{V}\:=\:\frac{\mathrm{n}\mathrm{u}\mathrm{m}\mathrm{b}\mathrm{e}\mathrm{r}\:\mathrm{o}\mathrm{f}\:\mathrm{t}\mathrm{r}\mathrm{u}\mathrm{e}\:\mathrm{n}\mathrm{e}\mathrm{g}\mathrm{a}\mathrm{t}\mathrm{i}\mathrm{v}\mathrm{e}\mathrm{s}}{\mathrm{n}\mathrm{u}\mathrm{m}\mathrm{b}\mathrm{e}\mathrm{r}\:\mathrm{o}\mathrm{f}\:\mathrm{t}\mathrm{r}\mathrm{u}\mathrm{e}\:\mathrm{n}\mathrm{e}\mathrm{g}\mathrm{a}\mathrm{t}\mathrm{i}\mathrm{v}\mathrm{e}\mathrm{s}+\mathrm{n}\mathrm{u}\mathrm{m}\mathrm{b}\mathrm{e}\mathrm{r}\:\mathrm{o}\mathrm{f}\:\mathrm{f}\mathrm{a}\mathrm{l}\mathrm{s}\mathrm{e}\:\mathrm{n}\mathrm{e}\mathrm{g}\mathrm{a}\mathrm{t}\mathrm{i}\mathrm{v}\mathrm{e}\mathrm{s}}$$

and

$$\:\mathrm{P}\mathrm{P}\mathrm{V}\:=\:\frac{\mathrm{n}\mathrm{u}\mathrm{m}\mathrm{b}\mathrm{e}\mathrm{r}\:\mathrm{o}\mathrm{f}\:\mathrm{t}\mathrm{r}\mathrm{u}\mathrm{e}\:\mathrm{p}\mathrm{o}\mathrm{s}\mathrm{i}\mathrm{t}\mathrm{i}\mathrm{v}\mathrm{e}\mathrm{s}}{\mathrm{n}\mathrm{u}\mathrm{m}\mathrm{b}\mathrm{e}\mathrm{r}\:\mathrm{o}\mathrm{f}\:\mathrm{t}\mathrm{r}\mathrm{u}\mathrm{e}\:\mathrm{p}\mathrm{o}\mathrm{s}\mathrm{i}\mathrm{t}\mathrm{i}\mathrm{v}\mathrm{e}\mathrm{s}+\mathrm{n}\mathrm{u}\mathrm{m}\mathrm{b}\mathrm{e}\mathrm{r}\:\mathrm{o}\mathrm{f}\:\mathrm{f}\mathrm{a}\mathrm{l}\mathrm{s}\mathrm{e}\:\mathrm{p}\mathrm{o}\mathrm{s}\mathrm{i}\mathrm{t}\mathrm{i}\mathrm{v}\mathrm{e}\mathrm{s}}\:.$$

## Supplementary Information

Below is the link to the electronic supplementary material.


Supplementary Material 1


## Data Availability

The datasets generated and/or analysed during the current study are available from the corresponding author on reasonable request.
