# Supplementary material for: Lateral Flow Assay to detect CXCL10 as a diagnose-supporting biomarker for Polyomavirus-associated Nephropathy (PVAN) after kidney transplantation (KTx)
Source: Sci Rep. 2026 Aug 27;16:26959. doi: 10.1038/s41598-026-66553-7 (PMC13522535; doi:10.1038/s41598-026-66553-7)
Supplement: Supplementary file 1 [file 41598_2026_66553_MOESM1_ESM.docx]

Lateral Flow Assay to detect CXCL10 as a diagnose-supporting biomarker for Polyomavirus-associated Nephropathy (PVAN) after kidney transplantation (KTx)

Lateral Flow Assay To Detect Polyomavirus-Associated Nephropathy

Emely Hauch^1^, Lisa K. Seiler^1^, Marianne Hoyer^1^, Irina Scheffner^2^, Wilfried Gwinner^2^, Patrick Lindner^1^, and Cornelia Blume^1^

^1^Institute of Technical Chemistry, Leibniz University Hannover, Callinstrasse 3-5, 30167 Hannover, Germany

^2^Department of Hypertensiology and Nephrology, Medical School Hannover, Carl-Neubert Strasse 1, 30625 Hannover, Germany

**Word count:** 3729 (without Abstract, References, Declaration of Interest)

**Correspondence information:**

Emely Hauch; Institut für Technische Chemie, Callinstr. 3-5, 30167 Hannover;
[emely.hauch@iftc.uni-hannover.de](mailto:emely.hauch@iftc.uni-hannover.de)

**OCID-IDs:**

Lisa K. Seiler 0000-0001-6287-5126

Emely Hauch 0009-0001-9013-2597

Irina Scheffner 0000-0002-5930-1866

Wilfried Gwinner 0000-0003-1703-893X

Cornelia Blume 0000-0001-7487-4884

**Supplement**

**Fig. S 1 Schematic drawing of a lateral flow assay to detect CXCL10 in different states.** (a) Major components of an LFA with CXCL10 as the analyte. (b) LFA with a positive test result. The analyte binds to the conjugate (detection antibody coupled to e.g., gold nanoparticles (AuNPs)) to make the signal visible in the test zone. The analyte-detection antibody-AuNP-complex migrates along the membrane and is bound by the capture antibody on the test zone. The remaining conjugate migrates to the control zone where the capture antibodies bind to the detection antibody of the conjugate. (c) LFA with a negative test result. Conjugate only binds in the control zone, indicating only a correct run-through of the sample. (d) Invalid test result, no conjugate is bound to the control zone

**Fig. S 2** **Regression analysis** of absolute urine CXCL10 concentrations in [pg/mL] and the CXCL10/urine creatinine ratio, with a regression coefficient of R^2^=0.89 (n=114, no concentration of creatinine available for five patients)

**Fig. S 3 Boxplots of CXCL10 urine concentrations in samples of KTRs of a protocol biopsy with a normal biopsy, urogenital infection (UTI), antibody-mediated rejection (ABMR), T cell-mediated rejection (TCMR), or polyomavirus-associated nephropathy (PVAN).** (a) rsp. absolute CXCL10 levels of these different entity groups plotted against the CXCL10/creatinine ratios (b). Diagram a) shows absolute concentration, and b) shows the CXCL10/creatinine ratio in urine samples of KTRs (no concentration of creatinine available for five patients). Asterisks indicate the level of significant differences between certain compared sample groups (*p<0.05, **p<0.01, ***p<0.001, ****p<0.0001 according to the Mann-Whitney-U-test, n. s. = not significant), with samples grouped according to their biopsy finding as indicated. Quartiles were calculated using the Tukey method. The boxes show the interquartile range (IQR) from Q1 (25th percentile) to Q3 (75th percentile), with the median (50th percentile) shown as a line within the box. The whiskers extend up to 1.5 times the IQR; outliers beyond this range are shown as individual data points. Means indicated in square points

**Fig. S 4 Receiver Operating Characteristic (ROC) Curves, describing the test performance of the newly developed lateral flow assay of patients with protocol biopsies**. The ROC curves comprise LFAs with samples from patients diagnosed with Polyomavirus-associated nephropathy (PVAN, n=17) according to BANFF criteria. (a) Samples from patients with no histological findings according to BANFF criteria (normal biopsy, n=43), (b) additionally with samples from patients with urinary tract infection (UTI, n=10), and (c) additionally with samples from patients with antibody-mediated rejection (ABMR, n=16). (d) 14 samples from patients with a T cell-mediated rejection (TCMR) were used

**Fig. S 5 Comparison of the Polyomavirus-associated nephropathy classes according to BANFF criteria and the associated CXCL10 concentrations.** The analysis was made using the Kruskal-Wallis ANOVA. The boxes show the interquartile range (IQR) from Q1 (25th percentile) to Q3 (75th percentile), with the median (50th percentile) shown as a line within the box. All data are shown as individual data points in the boxes. The medians of the three classes (class 1 n = 6, median = 73.54; class 2 n = 12, median = 18.99; class 3 n = 3, median = 191.86) did not differ significantly. There are no data available on the PVAN class for one patient

**Fig. S 6** **Receiver Operating Characteristic (ROC) Curves, describing the test performance of the newly developed lateral flow assay.** The ROC curve comprises LFAs with samples from patients diagnosed with Polyomavirus-associated nephropathy (PVAN, n=22) compared with (a) samples from patients with antibody-mediated rejection (ABMR, n=23), or compared with (b) samples from patients with T cell-mediated rejection (TCMR, n=18)

**Table S 1 CXCL 10 concentrations,** as detected with a standard ELISA test in urine samples, in different groups of KTRs, characterized by the protocol or indication biopsy finding, and according to the time after Tx in days

|  | **Protocol biopsy**  **~ 40 days** | **Protocol biopsy**  **~ 90 days** | **Protocol biopsy**  **~ 180 days** | **Protocol biopsy**  **~ 365 days** | **Indication biopsy**  **758 ± 741 days** |
| --- | --- | --- | --- | --- | --- |
| **Normal biopsy**  CXCL10 conc. (pg/mL)  Number of patient samples | 9.9 ± 8.7  9 | 23.5 ± 35.6  14 | 4.6 ± 5.4  13 | 3.1 ± 7.6  7 | 7.9 ± 6.8  2 |
| **TCMR**  CXCL10 conc. (pg/mL)  Number of patient samples | 188.0 ± 157.3  3 | 20.9 ± 20.9  6 | 8.6 ± 4.1  3 | 40.5 ± 39.2  2 | 15.7 ± 12.3  4 |
| **ABMR**  CXCL10 conc. (pg/mL)  Number of patient samples | 14.8 ± 9.7  3 | 8.9 ± 8.6  5 | 0.4 ± 0.0  1 | 17.9 ± 34.0  6 | 18.0 ± 19.1  8 |
| **PVAN**  CXCL10 conc. (pg/mL)  Number of patient samples | -  0 | 156.5 ± 85.5  6 | 133.1 ± 231.5  11 | -  0 | 24.4 ± 23.8  5 |
| **UTI**  CXCL10 conc. (pg/mL)  Number of patient samples | 8.5 ± 7.1  3 | 11.6 ± 15.2  5 | 19.7 ± 0.0  1 | 0.0 ± 0.0   1 | 45.4 ± 0.0  1 |

**Table S 2 Characteristics of patients with confirmed Polyomavirus-associated nephropathy with numbers.**  Number of patients of the three different Polyomavirus-associated nephropathy classes according to BANFF criteria, the corresponding BKV replications in serum at the day of biopsy and CXCL10 concentrations. There are no data available on the PVAN class for one patient

|  | **Number of patient samples** | **BKV-Replication**  **(*10^4^ copies/mL) in serum** | **CXCl10 concentration (pg/mL)** |
| --- | --- | --- | --- |
| **PVAN class 1** | 6/22 | 6.52 ± 19.7 | 73.54 ± 109.98 |
| **PVAN class 2** | 12/22 | 38.8 ± 168 | 18.99 ± 77.92 |
| **PVAN class 3** | 3/22 | 120 ± 65.1 | 191.86 ± 85.30 |
